# Effects of fumaric acid supplementation on methane production and rumen fermentation in goats fed diets varying in forage and concentrate particle size

**DOI:** 10.1186/s40104-018-0235-3

**Published:** 2018-02-09

**Authors:** Zongjun Li, Nannan Liu, Yangchun Cao, Chunjia Jin, Fei Li, Chuanjiang Cai, Junhu Yao

**Affiliations:** 1 College of Animal Science and Technology, Northwest A&F University, Yangling, Shaanxi 712100 China; 2 College of Pastoral Agricultural Science and Technology, Lanzhou University, Lanzhou, 730020 China

**Keywords:** Feed particle size, Fumaric acid, Goat, Methane, Ruminal fermentation

## Abstract

**Background:**

In rumen fermentation, fumaric acid (FA) could competitively utilize hydrogen with methanogenesis to enhance propionate production and suppress methane emission, but both effects were diet-dependent. This study aimed to explore the effects of FA supplementation on methanogenesis and rumen fermentation in goats fed diets varying in forage and concentrate particle size.

**Methods:**

Four rumen-cannulated goats were used in a 4 × 4 Latin square design with a 2 × 2 factorial arrangement of treatments: low or high ratio of forage particle size: concentrate particle size (Fps:Cps), without or with FA supplementation (24 g/d). Fps:Cps was higher in the diet with chopped alfalfa hay plus ground corn than in that with ground alfalfa hay plus crushed corn.

**Results:**

Both increasing dietary Fps:Cps and FA supplementation shifted ruminal volatile fatty acid (VFA) patterns toward more propionate and less acetate in goats. An interaction between dietary Fps:Cps and FA supplementation was observed for the ratio of acetate to propionate (A:P), which was more predominant when FA was supplemented in the low-Fps:Cps diet. Methane production was reduced by FA, and the reduction was larger in the low-Fps:Cps diet (31.72%) than in the high-Fps:Cps diet (17.91%). Fumaric acid decreased ruminal total VFA concentration and increased ruminal pH. No difference was found in ruminal DM degradation of concentrate or alfalfa hay by dietary Fps:Cps or FA. Goats presented a lower ruminal methanogen abundance with FA supplementation and a higher *B. fibrisolvens* abundance with high dietary Fps:Cps.

**Conclusions:**

Adjusting dietary Fps:Cps is an alternative dietary model for studying diet-dependent effects without changing dietary chemical composition. Fumaric acid supplementation in the low-Fps:Cps diet showed greater responses in methane mitigation and propionate increase.

## Background

In ruminants, between 2% and 12% of feed energy is lost in the form of methane (CH_4_) [1], which also contributes to global greenhouse gas emissions [2]. Increasing ruminal propionate production can not only decrease hydrogen available for methanogenesis but also increase the precursor available for gluconeogenesis in animals, improving feed efficiency [3, 4]. Therefore, the metabolic intermediates of the propionate-succinate pathway, such as malic acid and fumaric acid (FA), have received widespread attention in relation to their contribution to ruminal propionate production [5–7]. However, in many trials, no contribution to ruminal propionate production was observed [8, 9]. The conflicting results concerning responses to FA are probably attributable to diet-dependent effects: Yang et al. [5] and García-Martínez et al. [10] found that FA supplementation showed greater responses in high-forage diets than in low-forage diets. Nevertheless, the diet-related mechanism of FA is elusive due to differences among experiments in nutrient intakes or ruminal fermentation characteristics, especially dissolved hydrogen concentrations and microbiota, which could affect the conversion of FA to propionate [5, 11].

As illustrated by Janssen [12], dissolved hydrogen concentration in the rumen was higher in animals fed readily digestible feed. Adjusting the particle size of dietary forage and concentrate can influence their ruminal degradation kinetics due to changing surface area available for rumen microbes and enzymes [13–16], and subsequent alterations in rumen fermentation characteristics [15–18]; therefore, this way allows dietary effects to be investigated without changing chemical composition. Considering that balancing diets for physically effective NDF (peNDF) and rumen degradable starch (RDS) is a key to maintaining proper ruminal pH [19–21] and that improper ruminal pH can suppress ruminal degradation kinetics [20–22], a long particle size of alfalfa hay was used in a diet with a small particle size of corn in this study. Furthermore, the responses in CH_4_ emissions to dietary particle size remain unclear [23]. In addition, to minimize the confounding factors of animals’ feed intake and living environments, the experimental animals were limit-fed and kept in environmentally controlled chambers. The objective of this study was to investigate the effects of FA supplementation on CH_4_ production, ruminal fermentation, bacterial flora, and in situ feed degradation in goats that were fed diets that varied in forage and concentrate particle size.

## Methods

### Animals, diets, and experimental design

Experimental protocol was approved by the Animal Care and Use Committee of Northwest A&F University (Yangling, China). Four non-lactating Xinong Saanen dairy goats with an initial live weight of 50 ± 2.7 kg, approximately 4 yr of age, and with a permanent ruminal cannula were used. The goats were fed a ration that was formulated to meet the nutrient requirements for maintenance [24]. A total mixed ration (TMR) equivalent to 253 g alfalfa hay, 289 g corn silage, and 558 g concentrate (DM basis, Table 2) was offered to each goat twice daily in equal portions at 08:00 and 18:00 h. The goats had free access to drinking water. The amount of feed refused, if any, was recorded daily.

The goats were first allowed to adapt to the environmental chambers and the same TMR with chopped alfalfa hay and crushed corn over 4 wk, and then were assigned to a 4 × 4 Latin square design with a 2 × 2 factorial arrangement of treatments. The two factors were ratio of forage particle size to concentrate particle size (Fps:Cps, high or low) and FA supplementation (0 or 24 g/d). The dietary Fps:Cps was modulated by varying the methods used to process alfalfa hay (chopped or ground) and corn (crushed or ground). As shown in Tables 1 and 2, Fps:Cps was higher in the diet with chopped alfalfa hay (78% of particles > 8 mm) and ground corn (71% of particles < 1.18 mm) than in the diet with ground alfalfa hay (75% of particles < 8 mm) and crushed corn (74% of particles > 1.18 mm). FA (Aladdin Industrial Corporation Co., Ltd., Shanghai, China) was offered twice daily in equal with diet. The current study consisted of four experimental periods, and each period consisted of 15 d of adaptation to treatments and 11 d of sampling. A 7-d washout period between experimental periods was used to minimize potential carryover effects between experimental diets. During the washout period, goats were fed the same TMR with chopped alfalfa hay and crushed corn.

**Table 1:** The particle size distributions of alfalfa hay and corn using different processing method, % DM retained on sieve

Sieve size	Alfalfa hay		Sieve size	Corn	
	Chopped	Ground		Crushed	Ground
19.0 mm	31.8	0.7	2.36 mm	41.7	0
8.0 mm	46.5	24.3	1.18 mm	32.0	29.5
1.18 mm	13.2	45.2	0.55 mm	14.5	33.6
Pan	8.5	29.8	Pan	11.8	36.9

**Table 2:** Ingredients and chemical composition of experimental diets

Items	Treatment diet	
	Low-Fps:Cps	High-Fps:Cps
Ingredients, g/kg DM		
Crushed corn	370	–
Ground corn	–	370
Chopped alfalfa hay	–	230
Ground alfalfa hay	230	–
Corn silage	263	263
Cottonseed meal	40	40
Soybean meal	85	85
Premix^a^	5	5
CaHPO_4_	2.5	2.5
Salt	5	5
Particle, % of DM retained on sieve		
19.0 mm	14.8	19.8
8.0 mm	24.6	21.8
1.18 mm	39.0	27.4
Pan	21.6	31.0
Nutrient composition, % of DM		
DM	52.2	52.2
Crude protein	14.2	14.3
NDF	38.3	38.5
ADF	21.3	21.0
Starch	25.1	25.0
RDS	15.3	17.5
peNDF_1.18_	23.4	25.5
peNDF_1.18_/RDS	1.53	1.46

*peNDF* physically effective neutral detergent fiber, *RDS* rumen degradable starch, *Fps:Cps* ratio of forage particle size: concentrate particle size

^a^Premix (per kg) contains: Cu 370 mg, Fe 2200 mg, Zn 1800 mg, Mn 800 mg, I 30 mg, Se 30 mg, Co 50 mg, Vitamin A 200 kIU, Vitamin D_3_ 4500 IU, Vitamin E 6500 IU, Vitamin K_3_ 45 mg, Vitamin B_1_ 40 mg, Vitamin B_12_ 1 mg, Nicotinic acid 1,000 mg and Pantothenic acid 700 mg

Five indoor environmentally controlled chambers (each 7.4 m × 4.2 m × 2.7 m) were used in this study, with one chamber serving as an adaptation chamber and four chambers as gas measurement chambers. The four goats were housed in the adaptation chamber and separated by placing each in a metabolic cage (1.5 m × 1.0 m × 1.5 m), and they were moved to the gas measurement chambers with their cages only during the days when gas emissions were measured. The temperature inside the chambers was maintained between 22 and 26 °C, and the goats were subjected to a diurnal cycle of 14 h light and 10 h darkness throughout the experiment.

### Measuring daily methane production

The construction, operation and animal welfare of environmentally controlled chambers have been described in detail by Li et al. [25]. Briefly, the 24-h gas emissions from each goat were measured in 2 consecutive days (d 16 & 17, d 18 & 19) over 3 separate periods: 00:00 to 08:00 h and 18:00 to 24:00 h on the first day and then 08:00 to 18:00 h on the second day. At the end of each period, the chamber doors were kept open to allow fresh air in, and the chamber was cleaned before the next period. During the gas measurement periods, the air inside each chamber was mixed for 30 s every 10 min by 4 draft fans, and continuously and constantly pumped at a rate of 2 L/min to the CH_4_ analyzer and CO_2_ analyzer with infrared sensors (BAIF-Maihak Analytical Instrument Co., Ltd., China). The gases in the 4 chambers were analyzed sequentially, 5 min for each chamber in every 20 min.

The daily CH_4_ emission was calculated as follows:

$$ {\mathrm{C}\mathrm{H}}_4\kern0.28em \mathrm{emission}\left(\mathrm{L}/\mathrm{d}\right)=\Sigma \left[\left({\mathrm{C}}_{\mathrm{i}}-{\mathrm{C}}_{\mathrm{i}-1}\right)\times {\mathrm{V}}_{\mathrm{c}}+{\mathrm{V}}_{\mathrm{f}}\times \left({\mathrm{C}}_{\mathrm{i}}-{\mathrm{C}}_0\right)\right]/1000 $$

Where (C_i_ – C_i-1_) = the difference of CH_4_ concentration (mL/m^3^) in every 20 min; C_0_ = the initial CH_4_ concentration of each period; V_c_ = the chamber volume (83.9 m^3^); and V_f_ = the gas volume pumped from each chamber over each 20-min measurement (0.04 m^3^).

### Collection and analysis of ruminal fluid

Samples of ruminal fluid (over 80 mL) were collected through the cannula from the ventral rumen sac at three-hour sampling intervals on d 20 (07:00, 10:00, 13:00, 16:00 h), d 21 (08:00, 11:00, 14:00, 17:00 h) and d 22 (09:00, 12:00, 15:00, 18:00 h). For each rumen fluid sample, pH was measured immediately, and a 40-mL subsample and an 8-mL filtered subsample were stored at − 80 °C for later analyses of bacterial abundances and volatile fatty acid (VFA) concentrations, respectively. The filtered subsample was passed through four layers of gauze.

Ruminal VFA concentrations of each sample was analyzed by gas chromatography (Agilent Technologies 7820A GC system, Santa Clara, USA) using a 30 m × 0.25 mm × 0.33 μm fused silica column (AE-FFAP; ATECH Technologies Co., Ltd. China) after removing the solid particle and protein in the sample according to Li et al. [26].

The samples taken at 12 sampling times from the individual goats were thawed, and equal aliquots (1 mL) from each sample were composited into one sample, and then 200 μL of the composited sample was extracted total DNA using QIAamp DNA Stool Mini Kit (QIAgen, Valencia, CA) according to the manufacturer’s recommendation. The density and purification of the DNA samples were detected by gel electrophoresis and spectrophotometry (NanDrop 2000, Thermo Scientific, Madison, Wisconsin, USA). The 16S rDNA primers [27, 28] used to identify the methanogen, primary cellulolytic bacteria (*B. fibrisolvens*, *F. succinogenes* and *R. flavefaciens*) and fumarate-utilizing bacteria (*F. succinogenes* and *S. ruminantium*) in the rumen are listed in Table 3. The program and chemicals for quantitative real-time PCR assays were performed on a Bio-Rad iQ5 (Bio-Rad Laboratories, Hercules, CA, USA) according to Zhao et al. [29]. The relative population abundances of the specific bacteria were expressed as the percentages to the total bacterial 16S rDNA gene.

**Table 3:** PCR primers for real-time PCR assay

Target specie	Primer	Primer sequence (5′ to 3′)
Bacteria ^a^	Forward	CGGCAACGAGCGCAACCC
	Reverse	CCATTGTAGCACGTGTGTAGCC
Methanogen ^b^	Forward	GAGGAAGGAGTGGACGACGGTA
	Reverse	ACGGGCGGTGTGTGCAAG
*B. fibrisolvens* ^b^	Forward	GAGGAAGTAAAAGTCGTAACAAGGTTTC
	Reverse	CAAATTCACAAAGGGTAGGATGATT
*F. succinogenes* ^a^	Forward	GTTCGGAATTACTGGGCGTAAA
	Reverse	CGCCTGCCCCTGAACTATC
*R.flavefaciens* ^a^	Forward	CGAACGGAGATAATTTGAGTTTACTTAGG
	Reverse	CGGTCTCTGTATGTTATGAGGTATTACC
*S. ruminantium* ^b^	Forward	GGCGGGAAGGCAAGTCAGTC
	Reverse	CCTCTCCTGCACTCAAGAAAGACAG

^a^Denman and McSweeney, [27]; ^b^Khafipour et al. [28]

### In situ ruminal degradation

The in situ degradations of DM of the concentrate and alfalfa hay were performed according to NRC [30] on d 23 to 26. The concentrate and alfalfa hay were milled through a 2-mm screen. Subsamples of 5.0 g concentrate or 2.5 g alfalfa hay (DM basis) were placed in nylon bags (10 cm × 7 cm) with pore size of 50 μm. Bags were soaked in 37 °C water for 10 min before being inserted into the rumen. Bags with concentrate were taken out in duplicate after 2, 4, 8, 16, 24 and 48 h of the incubation, while bags with alfalfa hay were incubated in duplicate for 2, 4, 8, 16, 24, 48 and 72 h. Bags were washed clean under tap water and then analyzed for DM contents. The degradation parameters and effective ruminal degradability (ERD) were calculated according to the following model [31]:

$$ {\displaystyle \begin{array}{l}{\mathrm{Y}}_t=a+b\times \left(1-{\mathrm{e}}^{- kt}\right),\\ {}\mathrm{ERD}=a+b\times k/\left(k+ kp\right)\end{array}} $$

In which Y_*t*_ = disappearance proportion at time *t*, *a* = rapidly degradable fraction; *b* = slowly degradable fraction; *k* = constant rate of degradation of fraction *b*; *t* = time of incubation (h); *kp* = passage rate (0.04 /h for forage and 0.06 /h for concentrate [32]).

### Chemical analysis of feedstuff samples

Samples of the diets and orts were dried at 55 °C for 72 h and then ground through a 1-mm screen. Samples were analyzed for the content of DM, ash, crude protein according to AOAC [33], NDF and ADF according to Van Soest et al. [34] with sodium sulfite and heat stable α-amylase (Ankom® A200I fiber analyzer, ANKOM technology, Macedon, NY, USA).

Dietary peNDF content was calculated by the following formula [35]: peNDF = pef_1.18_ × NDF_1.18_, in which pef_1.18_ represents the percent weight (DM basis) of particles retained above the 1.18-mm sieve of the Penn State Particle Separator [15], and NDF_1.18_ is the NDF content of particles retained on the 1.18-mm sieve.

Dietary RDS content was calculated by the following formula [36]: RDS = Σ *P*_*i*_ × ERD_*i*_, in which *P*_*i*_ represents the proportion of starch content of feed *i* in the diet, ERD_*i*_ represents starch ERD of feed *i*, which included concentrate, alfalfa hay and corn silage, and were measured twice before and after feeding experiment. The starch content of feed and samples of in situ degradation was measured by a commercial Total Starch Assay kit (Megazyme, International Ireland Ltd., Bray, Co. Ireland).

### Data analysis

The data were analyzed using the MIXED procedure of SAS 9.2 (SAS Institute Inc., Cary, NC); Fps:Cps, FA, and their interaction were fixed factors, and experimental period and animal were random factors. When there was an interaction, Tukey’s multiple comparison tests were used to assess differences among treatment means.

The dynamics of ruminal pH after the morning feeding were analyzed as a one-way repeated measures ANOVA using the PROC MIXED program in SAS; Fps:Cps, FA, and their interaction were fixed factors, experimental period and animal were random factors, and sample time was the repeated factor. The dynamics of ruminal pH after the morning feeding on d 21 were different with the other 2 d, due to sampling stress at feeding time (08:00 h) resulting in a much longer eating duration; therefore, the results of ruminal pH at 08:00, 11:00, 14:00, 17:00 h on d 21 and 18:00 h (afternoon feeding) on d 22 were not included in this analysis. The dynamics of ruminal pH after the morning feeding were performed separately for Fps:Cps and FA in figure, as no interaction between them was observed at any time point.

The probability level was set at *P* < 0.05 for significance and 0.05 ≤ *P* < 0.10 for trend.

## Results

### Methane production

On average, the daily dry matter intake (DMI) was 1.08 kg per goat that over 95% of the diets offered was consumed. No difference (*P* > 0.1) in DMI was observed among treatments. Fumaric acid supplementation decreased CH_4_ production (*P* < 0.05) in goats, expressed as L/d or as L/kg DMI, independently of the dietary Fps:Cps (Table 4). Although the interaction between dietary Fps:Cps and FA was not significantly related to CH_4_ production, the reduction in CH_4_ production with FA was greater in the low-Fps:Cps diet (31.72%) than in the high-Fps:Cps diet (17.9%).

**Table 4:** Effects of dietary Fps:Cps and FA supplementation (0 or 24 g/d) on CH_4_ emissions and ruminal fermentation parameters

Items	Low-Fps:Cps		High-Fps:Cps			*P*-value		
FA, g/d	0	24	0	24	SEM	Fps:Cps	FA	(Fps:Cps) × FA
CH_4_, L/d	23.14	15.80	22.84	18.75	1.245	0.552	0.021	0.466
CH_4_/DMI, L/kg	21.03	14.54	20.94	17.95	1.044	0.365	0.020	0.341
pH	6.38	6.52	6.40	6.51	0.027	0.887	0.020	0.817
Total VFA, mmol/L	89.19	85.89	87.01	80.53	1.326	0.633	< 0.001	0.187
Individual VFA molar proportion, %								
Acetate	70.24	65.50	66.93	63.50	0.526	0.009	< 0.001	0.518
Propionate	14.80	20.58	19.07	21.98	0.437	0.001	< 0.001	0.074
Butyrate	11.93^a^	10.79^b^	10.82^b^	11.20^ab^	0.170	0.303	0.259	0.026
A:P	4.89^a^	3.62^b^	3.74^b^	3.15^c^	0.095	< 0.001	< 0.001	0.048

*FA* fumaric acid, *Fps:Cps* ratio of forage particle size: concentrate particle size, *A: P* Acetate: propionate, *SEM* pooled standard error of the means

^a,b^Different letters in the same row means significant differences (*P* < 0.05)

### Ruminal fermentation characteristics

The effects of dietary Fps:Cps and FA on ruminal fermentation parameters are shown in Table 4. FA supplementation decreased the ruminal total VFA concentration (*P* < 0.01) and raised pH (*P* < 0.05), independently of the dietary Fps:Cps. Both increasing dietary Fps:Cps and FA supplementation reduced (*P* < 0.01) the ruminal acetate proportion and the ratio of acetate to propionate (A:P), and increased (*P* < 0.01) propionate proportion. An interaction between dietary Fps:Cps and FA was observed for the A:P (*P* < 0.05), and a tendency was observed for the propionate proportion (*P* = 0.07). FA increased ruminal propionate proportion by 39% in the low-Fps:Cps diet and by 15% in the high-Fps:Cps diet, and decreased the A:P by 26% in the low-Fps:Cps diet and by 16% in the high-Fps:Cps diet. In addition, an (Fps:Cps) × FA interaction effect also occurred (*P* < 0.05) for butyrate proportion, which was reduced (*P* < 0.05) by FA in the low-Fps:Cps diet but not affected in the high-Fps:Cps diet.

**Fig. 1:**
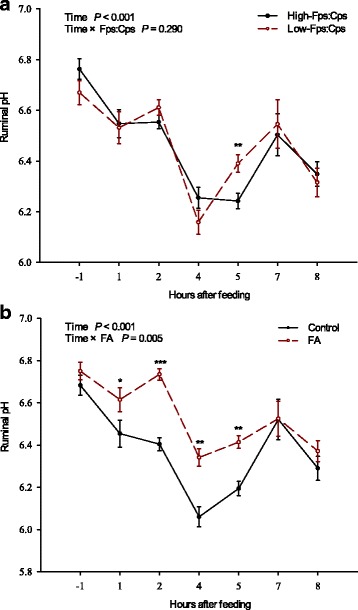
Effects of dietary Fps:Cps (**a**) and FA (**b**) on the dynamics of ruminal pH after the morning feeding in goats. FA = fumaric acid; Fps:Cps = ratio of forage particle size: concentrate particle size. *0.05 ≤ *P* < 0.1; **P < 0.05; ***P < 0.01

Ruminal pH dropped after feeding (*P* < 0.001), with a nadir at 4^th^ postprandial hour in all groups, and increased thereafter (Fig. 1). There was no (Fps:Cps) × FA interaction at any sample time point (*P* > 0.1). The dynamics of ruminal pH after the morning feeding between low- and high-Fps:Cps diet was consistent (*P* > 0.1), except that the ruminal pH was higher at the 5^th^ postprandial hour in the low-Fps:Cps diet than in the high-Fps:Cps diet (Fig. 1a). Fumaric acid supplementation attenuated the ruminal pH drop after feeding, and the attenuation was dependent on time (*P* < 0.01), as the ruminal pH increased (*P* < 0.05) with FA from the 2^nd^ to the 5^th^ postprandial hours and tended to increase (*P* = 0.08) at the 1^st^ postprandial hour.

### Ruminal bacterial population abundances and in situ degradation of DM

Fumaric acid supplementation reduced the abundance of methanogens (*P* < 0.05, Table 5) in the rumen. The abundance of *B. fibrisolvens* was lower in goats fed the low-Fps:Cps diet than the high-Fps:Cps diet (*P* < 0.05). No differences in the abundances of *F. succinogenes*, *R. flavefaciens*, or *S. ruminantium* were observed among treatments.

**Table 5:** Effects of dietary Fps:Cps and FA supplementation (0 or 24 g/d) on the relative abundances of rumen bacteria

Items	Low-Fps:Cps		High-Fps:Cps			*P*-value		
FA, g/d	0	24	0	24	SEM	Fps:Cps	FA	(Fps:Cps) × FA
Total bacterial abundance, × 10^−4^								
Methanogens	15.11	12.37	15.84	12.67	0.699	0.711	0.036	0.875
*F. succinogenes*	89.26	86.72	81.61	91.17	4.493	0.861	0.703	0.511
*R. flavefaciens*	2.10	2.03	1.81	2.14	0.121	0.725	0.611	0.443
*S. ruminantium*	8.21	8.72	7.79	8.36	0.261	0.467	0.312	0.965
*B. fibrisolvens*	4.07	3.87	5.36	4.84	0.233	0.014	0.408	0.711

*FA* fumaric acid, *Fps:Cps* ratio of forage particle size: concentrate particle size, *SEM* pooled standard error of the means

Data are expressed as a fraction of the total bacterial 16S rDNA

Neither dietary Fps:Cps nor FA had any impact on ruminal degradation kinetic parameters or on the ERD of the concentrate or alfalfa hay DM in goats (*P* > 0.1, Table 6).

**Table 6:** Effects of dietary Fps:Cps and FA supplementation (0 or 24 g/d) on in situ DM degradability

Diets	Low-Fps:Cps		High-Fps:Cps			*P*-value		
FA, g/d	0	24	0	24	SEM	Fps:Cps	FA	Fps:Cps × FA
Concentrate DM, %								
*a*	11.83	13.77	10.18	11.19	2.315	0.715	0.749	0.959
*b*	91.09	85.21	84.09	84.75	2.491	0.498	0.634	0.551
*k*, /h	4.95	5.98	6.43	6.01	0.283	0.194	0.586	0.209
ERD	68.16	69.35	67.37	67.74	1.256	0.674	0.781	0.884
Alfalfa hay DM, %								
*a*	16.18	15.00	18.39	15.08	1.647	0.755	0.545	0.773
*b*	37.95	41.19	41.06	39.04	1.164	0.850	0.808	0.310
*k*, /h	11.45	11.12	8.54	13.42	1.545	0.927	0.505	0.446
ERD	47.45	48.67	51.26	47.08	0.970	0.560	0.446	0.181

*FA* fumaric acid, *Fps:Cps* ratio of forage particle size: concentrate particle size, *SEM* pooled standard error of the means

*a* = rapidly degradable fraction, *b* = slowly degradable fraction, *k* = constant rate of degradation of fraction b; ERD = effective ruminal degradability

## Discussion

### Dietary model of Fps:Cps and its effects on methane production and rumen fermentation

In this study, we established a dietary model of Fps:Cps based on the dietary forage: concentrate ratio. Increasing dietary forage: concentrate ratio and decreasing dietary Fps:Cps represented offering more forage and less concentrate available for ruminal microbes and enzymes, in amount and in surface area, respectively. Adjusting dietary Fps:Cps altered the dietary physical characteristics without changing the dietary chemical composition but also changed the dietary fermentation characteristics in the rumen such that the A:P was lower in goats fed the high-Fps:Cps diet. Consistently, both increasing the forage particle size and decreasing the concentrate particle size shifted ruminal VFA patterns toward lower A:P in previous studies [15, 17, 18]. However, neither the ruminal total VFA concentration nor in situ degradations of the concentrate or alfalfa hay DM were affected by dietary Fps:Cps in this study, indicating that the dietary Fps:Cps manipulated ruminal fermentation pathways mainly by shifting availability of feedstuff surface area [13, 16, 19] rather than shifting the capacity of ruminal degradability. In contrast, Plaizier et al. [37] found that ground wheat and barley decreased the ruminal degradability of mixed hay DM and NDF in dairy cows with a lower average ruminal pH of 5.87. However, the pH optima for ruminal protozoa, primary cellulolytic bacteria and most secondary bacteria are 6.2 or higher [22]. Furthermore, considering the negative correlation between ruminal degradation and pH [38, 39], the absence of changes in ruminal degradation kinetic parameters or ERD was partly attributed to the similar diurnal fluctuations in rumen pH between low- and high-Fps:Cps diets. It is commonly believed that balancing the amount of dietary peNDF and RDS is a key to manipulating ruminal pH and maintaining proper rumen metabolism [19–21]. The ratios of peNDF to RDS were 1.53 and 1.46 in the low-Fps:Cps diet and the high-Fps:Cps diet, respectively, higher than the ratio of 1.43 suggested by Li et al. [26] to lower the risk of subacute ruminal acidosis in dairy goats.

Fermentative acetate production during rumen fermentation is accompanied by hydrogen production, whereas propionate production is accompanied by hydrogen consumption [12, 40]. However, in the current experiment, increasing dietary Fps:Cps had no effect on CH_4_ production, although it decreased the ruminal A:P. Based on the pathways of carbohydrate fermentation, the hydrogen available for methanogenesis depends not only on the ruminal VFA profiles but also on the amount of dietary carbohydrate fermented. As illustrated by Janssen [12], dissolved hydrogen concentration in the rumen was higher in animals fed readily digestible feed; therefore, hydrogen-suppression with low ruminal A:P in the goats fed high-Fps:Cps diet was compensated by more RDS fermented in the rumen. In addition, adjusting dietary Fps:Cps might affect the methanogenesis by influencing digesta passage, animal chewing and ruminating activities, and ruminal motility [21, 23] besides by influencing ruminal VFA profiles and degradation kinetics; however, these pathways might also cancel each other out in this study. Hironaka et al. [41] observed that reducing the particle size of alfalfa hay had no effect on CH_4_ emissions in steers, either. Consequently, further studies are required to better understand the relationships among those metabolic pathways on methanogenesis.

### Fumaric acid and its relationships with dietary Fps:Cps

Fumaric acid supplementation altered the ruminal fermentation pathways toward more propionate production and lower CH_4_ emissions, agreeing with previous studies [5–7]. Theoretically, conversion of all 24 g FA (0.21 mol) to propionate could potentially reduce CH_4_ production by 1.27 L/d (FA: hydrogen: CH_4_ = 4 mol: 4 mol: 1 mol, 24.5 L/mol CH_4_), which is much lower than the actual reduction (mean of 6.49 L/d in the low-Fps:Cps diet and 4.09 L/d in the high-Fps:Cps diet) in this study. Yang et al. [5] and van Zijderveld et al. [42] also observed that the actual reduction in CH_4_ production with FA was greater than the potential reduction, which indicated that the CH_4_-suppressing effects of FA were not only attributable to its function as an alternative electron-acceptor to methanogenesis. Although the abundances of fumarate-utilizing bacteria (*F. succinogenes* and *S. ruminantium*) were not affected by FA in this study, FA likely stimulated fumarate-utilizing bacteria and thus increased the ruminal activity of the succinate-propionate metabolic pathway [5, 43], which stimulated other substrates (such as starch and fiber) in feed to be fermented as propionate. Moreover, the contributions to propionate increase with FA by the stimulation of fumarate-utilizing bacteria may be larger than the conversion of FA itself. The increase in ruminal propionate production with FA would have a starvation effect on methanogens, such as hydrogenotrophic archaea, reducing their abundance, which agrees with our results and those of a previous study [5].

In the current study, FA supplementation to the low-Fps:Cps diet showed greater responses in CH_4_ mitigation and propionate increase. Considering that the hydrogen affinity of FA is lower than that of methanogenesis [44–46], high dissolved hydrogen concentrations in the rumen would accelerate the conversion of FA to propionate according to the thermodynamic principle [11]. Dissolved hydrogen concentrations in the rumen was higher in animals fed readily digestible feed [12, 47]. Therefore, the metabolism of FA was slower in the animals fed low-Fps:Cps diet than fed high-Fps:Cps diet, and the duration of the positive stimulation of FA on fumarate-utilizing bacteria became longer, resulting in greater responses in CH_4_ mitigation and propionate increase. This suggests that the responses to FA depend on the rumen’s capacity to metabolize FA, rather than the direct interactions between FA and dietary nutrients. This theory also explained why FA supplementation in high-forage diets showed greater responses than that in low-forage diets [5, 10]. The abundances of fumarate-utilizing bacteria (*F. succinogenes* and *S. ruminantium*) in the rumen between low- and high-Fps:Cps diets were not different; however, they can also influence the rumen’s capacity to metabolize FA. Therefore, more studies are needed to fully understand the relationship between fumarate-utilizing bacteria abundances and ruminal responses to FA.

To our knowledge, little information is available regarding the effects of FA on in situ degradation of feedstuff. In the present study, the in situ degradability of DM in the concentrate and alfalfa hay were not affected by FA supplementation, but interestingly, the ruminal total VFA concentration was reduced. Considering that ruminal pH is negatively related to total VFA concentration [48], an increase in ruminal pH with FA would be expected, consistent with that of previous studies [9, 49]. An alternative explanation to the higher ruminal pH might be that FA stimulated fumarate-utilizing bacteria to uptake lactic acid [9, 50]. Inconsistently, Remling et al. [51] reported that adding FA (300 or 600 g) to diets for cattle decreased the rumen pH, and Vyas et al. [8] and McGinn et al. [52] reported no changes in ruminal pH with FA supplementation. Thus, the effects of FA supplementation on ruminal pH need to be further confirmed. In addition, the effect of FA on increasing ruminal pH was dependent on time after feeding, as FA increased the pH during the first 5 h after feeding but had no effect thereafter, indicating the duration of FA metabolization in the rumen.

## Conclusion

Dietary Fps:Cps is an alternative dietary model to dietary forage-to-concentrate ratio for studying diet-dependent effects, and it could manipulate ruminal fermentation patterns without the confounding effect of varying dietary nutrient composition. Increases in propionate with FA were attributed to its conversion to propionate and its positive stimulation of fumarate-utilizing bacteria, resulting in the actual CH_4_-depressing effects of FA being greater than the anticipated effects. The responses to FA supplementation likely depend on the rumen’s capacity to metabolize FA, with the low-Fps:Cps diet showing greater responses.

## Abbreviations

A:P: Acetate to propionate ratio
ADF: Acid detergent fiber
DM: Dry matter
ERD: Effective ruminal degradability
FA: Fumaric acid
Fps:Cps: Ratio of forage particle size: concentrate particle size
NDF: Neutral detergent fiber
peNDF: Physically effective neutral detergent fiber
RDS: Rumen degradable starch
VFA: Volatile fatty acids
